# A machine-learning approach to a mobility policy proposal^☆^

**DOI:** 10.1016/j.heliyon.2023.e20393

**Published:** 2023-09-27

**Authors:** Miljana Shulajkovska, Maj Smerkol, Erik Dovgan, Matjaž Gams

**Affiliations:** Jožef Stefan Institute, Jamova cesta 39, SI-1000 Ljubljana, Slovenia

**Keywords:** Machine learning, Smart cities, Mobility policy

## Abstract

The objective of the URBANITE project is to design an open-data, open-source, smart-city framework to enhance the decision-making processes in European cities. The framework's basis is a robust and user-friendly simulation tool that is supplemented with several innovative service modules. One of the modules, a multi-output, machine-learning unit, is deployed on the simulation results, enabling city officials to more effectively analyse vast quantities of data, discern patterns and trends, and so facilitate advanced policy decisions. The city's decision makers define potential city scenarios, key performance indicators, and a utility function, while the module assists in identifying the policy that is best aligned with the stipulated constraints and preferences. One of the main improvements is a speeding up of the policy testing for the decision makers, reducing the time needed for one policy verification from 3 hours to around 10 seconds. The system was evaluated for Bilbao's Moyua area, where it suggested strategies that could result in a decrease in emissions of more than 5% $CO_{2}$, *NOx*, *PM* in the selected area and a broader part of the city with a machine-learning accuracy of 91%. The system was therefore able to provide valuable insights into effective policies for restricting private traffic in specific districts and identifying the most advantageous times for these restrictions.

## 1 Introduction

The rapid growth in the world's population combined with a trend for increasing urbanisation presents modern cities with challenges. According to the United Nations, the global population is projected to increase by nearly 2 billion people in the next 30 years, with 7 out of 10 individuals residing in urban areas [1]. To address the issues associated with rapid urbanisation and achieve sustainable development, the concept of smart cities (SCs) has emerged. Numerous studies have explored the definitions of SCs [2], [3], [4], [5], and recent advances in technology have empowered the development of SCs to enhance urban performance and improve people's quality of life. In particular, the utilisation of Information and Communication Technologies (ICTs) in the latest research has enabled the implementation of smart strategies and the delivery of information to diverse users within SCs [6], [7], [8]. In this context, recent advances in artificial intelligence (AI) play a crucial role in the intelligent management of SCs. The availability of vast amounts of data and the application of AI algorithms help decision makers when they are designing, selecting and implementing various services within SCs.

One critical aspect of SCs is the design and implementation of mobility policies that cater to the subjective preferences of a city's residents. However, many mobility policies, such as infrastructure improvements, involve significant costs, and each modification must be carefully evaluated to assess its impact on traffic flow. Microscopic traffic simulators have become valuable tools for evaluating these policies in advance [9], [10]. Simulating different scenarios requires the utilisation of data related to a map of the city, people's movements, and other mobility information. Typically, expert knowledge is involved in evaluating the performance of simulated mobility policies. Nevertheless, testing numerous infrastructure and mobility configurations can be time consuming and challenging when trying to identify the most effective options.

**Figure 1:**
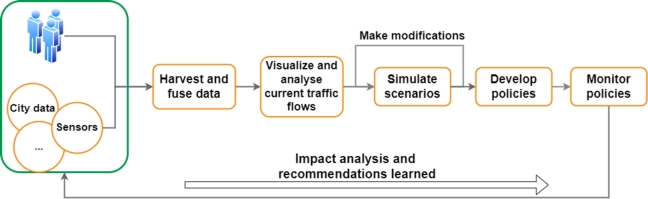
Traditional smart-city decision-making process.

The traditional approach to evaluating mobility policies follows a systematic process, as illustrated in Fig. 1. City managers begin by gathering and analysing diverse data sources, including real-time traffic data and demographic information, to gain a comprehensive understanding of the existing transport landscape. They then engage with stakeholders to develop policies that consider the diverse needs and preferences of the city's residents. Decision makers employ policy-evaluation frameworks to assess various options based on multiple criteria such as congestion, sustainability and economic impact. Through scenario analysis and simulation models, they project the potential outcomes and impacts of each policy option, enabling data-driven decision making (DM). Once a policy is selected, city managers focus on its implementation by coordinating with relevant departments, agencies and service providers. They ensure proper execution, closely monitor progress and make necessary adjustments to optimise policy outcomes. The continuous evaluations of implemented mobility policies are crucial, relying on ongoing data analysis, performance metrics, and stakeholder feedback to assess their impact on transport systems, environmental sustainability and social well-being. Traditional decision-making processes in mobility policy, therefore, involve simulating numerous scenarios to evaluate their outcomes and select the best option. However, this approach can be resource intensive and time consuming.

Machine-learning (ML) techniques have been successfully applied in many automation tasks, but rarely in the domain of SCs. The idea is to use ML to enhance urban-mobility planning by enabling policymakers and public servants to make informed decisions [11]. Instead of simulating every possible scenario, the ML model can be trained on a subset of pre-simulated scenarios. By leveraging the existing dataset, the ML-generated model can generate policy recommendations effectively without the need for an extensive simulation. This ML approach could then reduce the time and resources required for decision making, while maintaining reliable policy suggestions.

The major research hypothesis is that multi-output ML methods can be applied to the simulations' output to automate the decision making and speed up the optimisation.

By allowing decision makers to bypass the time-consuming simulation step and directly predict the best scenario by specifying the desired output of a mobility policy, such a system enhances the efficiency, accuracy and consistency of the decision making and eliminates any subjective biases that might arise from a manual analysis. Furthermore, the proposed ML approach enables the use of subjective key performance indicators (KPIs) and provides other ML advantages.

The specific challenge addressed in this study is the development of a ML approach for optimising a city's mobility-policy proposals in Bilbao. By leveraging data from microscopic traffic simulators, this approach utilises data-analysis techniques to significantly expedite the decision-making process. Instead of simulating all the possible scenarios, the ML model is trained on a subset of those possible scenarios, employing a single simulation run as a training-data instance. The feature vectors are defined based on KPIs that describe the desired changes in the city. By doing so, the ML model can propose effective solutions, even when facing a large number of potential infrastructure changes. This ML methodology assists decision makers in making well-informed choices when optimising mobility in a city and providing several additional advantages of ML such as transparency and an in-depth analysis.

The study and the application in Bilbao constitute part of the URBANITE project [12], which aims to design a long-term, sustainable, ecosystem model supported by a data platform and algorithms [13], [14], [15], [16], [17]. Leveraging this analysis, the algorithms develop recommendations for mobility policies that improve specific KPIs defined for each of the four cities: Bilbao, Amsterdam, Messina and Helsinki. The URBANITE project demonstrates the critical role of ML algorithms in processing and analysing vast amounts of data to provide valuable insights into mobility planning for cities.

The paper's structure is as follows. First, we described the methods used for developing the proposed system. Then, information about the data and the results obtained in this study is discussed. The final section presents conclusions and suggestions for future research.

## 2 Methodology

### 2.1 General schema of the proposed approach

**Figure 2:**
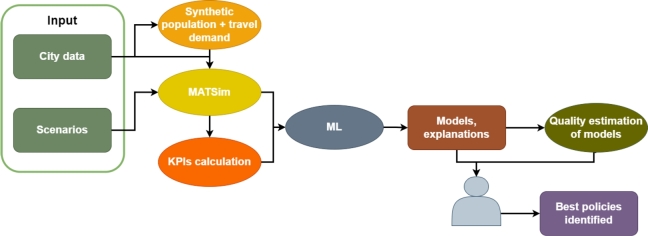
System structure.

The proposed approach follows the general schema outlined in Fig. 2. It begins by collecting the city data necessary for the synthetic population and the travel-demand models. Once this data is generated, it is fed into the MATSim simulator, which can be configured and run. Numerous simulation scenarios will produce a vast amount of data that can be analysed to identify patterns. Part of this data is used to calculate KPIs for the city. The output from the simulator, along with the KPIs, is used to train the ML models. A ML module can be used to create models and provide explanations for end-users. Additionally, statistical methods can be employed to evaluate the quality of the models. Finally, the end user is equipped to determine the most appropriate policies for implementation. In the subsequent sections, the data and outcomes are analysed and discussed.

### 2.2 Constructing simulated populations and modelling travel demand

Microscopic traffic simulators employ artificial agents to simulate large populations, requiring a modelled travel demand for a realistic traffic analysis. An alternative involves generating a synthetic population that mirrors the actual demographics, coupled with daily plans through origin-destination matrices.

The majority of population synthesisers expand a representative sample of the people represented as agents, i.e., a dis-aggregated dataset, using marginal distributions, i.e., an aggregated dataset, into a fully enumerated census [18]. The sample or dis-aggregated data comprises a list of individuals with demographic attributes, while the aggregated data contains the demographics of a small geographical area for which the synthetic population should be generated. The sample data typically do not cover the entire population. To solve this issue, a wide range of methods exist, which integrate both the aggregated and dis-aggregated datasets and reconstruct the entire population. The iterative proportional fitting (IPF) algorithm [19] is one of the most widely used deterministic algorithms for synthetic-population generation. It is used to adjust the population data to ensure consistency between the marginal distributions of auxiliary information, such as age, gender and other demographic variables. The IPF method adjusts the data iteratively to achieve the best solution within the constraints of the auxiliary information.

The travel-demand model is essential for sustainable city planning, leveraging synthetic population data and activity locations to create the travel demand. An accurate demand determination is the key to assessing the various mobility strategies and policies.

### 2.3 Simulation framework MATSim

Once the travel demand is created, the traffic situation in the city is modelled and the effects of a particular mobility policy are analysed. For this purpose, a traffic micro-simulation tool is used. Several state-of-the-art solutions were tested and MATSim was chosen as the most suitable. MATSim [20] is an activity-based, multi-agent, open-source tool implemented in Java. It simulates the traffic behaviour of individuals as well as improving it, i.e., making it similar to real-life traffic. The behaviour of the traffic is improved by a co-evolutionary optimisation.

**Figure 3:**
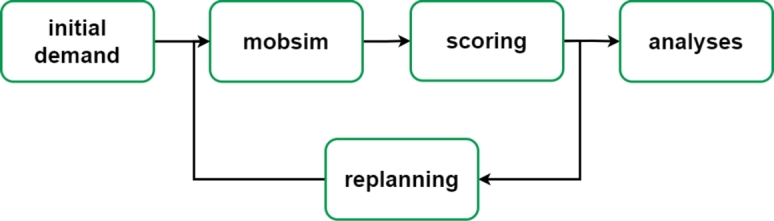
MATSim steps.

The MATSim simulator requires several input files related to the travel demand, city map, public-transit schedules, vehicle type, etc. The simulation steps are shown in Fig. 3, with the simulation being optimised in several iterations. The first iteration starts with an initial travel demand from the study area. The individuals, called agents in MATSim, store a fixed number of plans, where each plan contains a list of daily activities (e.g., home, work, leisure) and a score. Each agent learns by maintaining multiple plans, which are scored by executing them in the mobsim, selected according to the score and modified if necessary. The plan can be modified using the replanning module, where four dimensions are considered, related to the departure time, route, mode and destination. The iterative process is concluded when the average population score converges.

The scoring module evaluates the performance of the plan in a synthetic reality and updates the agents' plan for the next iteration [21]. Next, only the plans with the highest scores are selected by the agent, while the others are deleted in the replanning step. The scores are computed using a scoring function that takes into account the performance of activities and the travel time. A typical score is calculated as follows:

(1) $$S_{\mathrm{plan}}=\sum_{q=0}^{N-1}S_{\mathrm{act},q}+\sum_{q=0}^{N-1}S_{\mathrm{trav},\mathrm{mode}(q)}$$

The utility of a plan $S_{\mathrm{plan}}$ (Equation (1)) is the sum of all the activity utilities $S_{\mathrm{act},q}$ plus the sum of all the travel utilities $S_{\mathrm{trav},\mathrm{mode}(q)}$. *N* represents the number of activities. The utilities for the activity *q* and the travel are calculated as follows:

(2) $$S_{\mathrm{act},q}=S_{\mathrm{dur},q}+S_{\mathrm{wait},q}+S_{\mathrm{late}.\mathrm{ar},q}+S_{\mathrm{early}.\mathrm{dp},q}+S_{\mathrm{short}.\mathrm{dur},q}$$

(3) $$S_{\mathrm{trav},q}=C_{\mathrm{mode}(q)}+\beta_{\mathrm{trav},\mathrm{mode}(q)}t_{\mathrm{trav},q}+\beta_{m}\Delta m_{q}+(\beta_{d,\mathrm{mode}(q)}+\beta_{m}\gamma_{d,\mathrm{mode}(q)})d_{\mathrm{trav},q}+\beta_{\mathrm{transfer}}x_{\mathrm{transfer},q}$$

The activity utility $S_{\mathrm{act},q}$ (Equation (2)) sums up the duration $S_{\mathrm{dur},q}$ and the waiting time $S_{\mathrm{wait},q}$ for an activity to start, a penalty for a late arrival $S_{\mathrm{late}.\mathrm{ar},q}$ and an early departure $S_{\mathrm{early}.\mathrm{dp},q}$ at the activity location, and a penalty for the short duration of an activity $S_{\mathrm{short}.\mathrm{dur},q}$. The travel utility $S_{\mathrm{trav},q}$ (Equation (3)) depends on the mode-specific constant $C_{\mathrm{mode}(q)}$, the marginal utility $\beta_{\mathrm{trav},\mathrm{mode}(q)}$ of the time spent travelling between two activity locations by mode $t_{\mathrm{trav},q}$, the marginal utilities of money $\beta_{m}$, distance $\beta_{d,\mathrm{mode}(q)}$ and public-transfer penalties $\beta_{\mathrm{transfer}}$, the monetary budget resulting from the fares $\Delta m_{q}$ and mode-specific monetary distance rate $\gamma_{d,\mathrm{mode}(q)}$, the distance travelled between two locations $d_{\mathrm{trav},q}$, and whether a transfer has occurred between the previous and the current leg $x_{\mathrm{transfer},q}$ (Boolean variable).

Several files are produced as the output of the simulation. These can be used for different analyses. Some of them refer to one iteration and some summarise a complete simulation run. The results are used to compute the KPIs for the ML models, as described in the following sections.

### 2.4 Key performance indicators

KPIs are measurable values that reflect the progress of the city in achieving its goals. They are an important tool for city decision makers as they provide a clear and quantifiable way to evaluate the impact of their policies and strategies. KPIs help decision makers understand how well they are performing in relation to their objectives and identify areas where they need to improve. Examples of KPIs in the context of traffic and transport include measures for traffic congestion, air quality, travel time, etc. The KPIs can be used to evaluate the effectiveness of different mobility strategies, such as the implementation of bike lanes, public-transport systems, car-sharing programmes or traffic restrictions. By regularly monitoring KPIs, decision makers can adjust their strategies as necessary to achieve their goals and build a more sustainable city.

While KPIs are usually computed in the form of an equation, they normally represent an objective variable such as the amount of $CO_{2}$ or a subjective preference function of one or several variables. A subjective preference would be that lowering $CO_{2}$ emissions is more important than reducing the impact of traffic jams.

### 2.5 Multi-output machine learning for suggesting mobility policies

The developed ML-based approach aims at supporting decision makers in their selection of a mobility policy. It learns from the available microscopic traffic simulations and then predicts which policy actions should be applied in order to obtain the preferred objective values. The input in the form of the obtained simulation is considered as the ground truth, i.e., one that needs no validation.

The policy-prediction model is trained as follows. The objectives, i.e., the KPI values of the simulations, are treated as features, while the policies, i.e., the scenarios, are treated as target variables. An example of a scenario would be: “Close Moyua square from 17h to 19h.” This enables the decision makers to select the preferred KPI values, which are then used by the policy-prediction model to predict the most suitable scenario or mobility policy.

Because of the complexity of the problem and the heterogeneous nature of the target variables, multi-output algorithms were applied in the form of multi-label classification and multi-output regression.

Dealing with complex problems in SC decision making has led to the use of multi-output learning, because multi-label classification algorithms try to predict multiple discrete variables simultaneously [22]. The problem can be formulated as follows. Let *D* be the training dataset containing *N* $E_{i}=(X_{i},Y_{i})$ examples, where i=1,...,N. Each instance $E_{i}$ is associated with a feature vector $X_{i}=(x_{i1},x_{i2},...x_{im})$ and a subset of $Y_{i}\subseteq L$ where $L=\{y_{j}:j=1...q\}$ is the set of *q* possible labels. The task is to create a classifier *H* that will accurately predict an unlabelled instance *E* described with the feature vector *x* and an undefined subset of labels *Y*, i.e., H(E)−>Y.

One approach to achieving multi-label classification involves using transformation techniques that support this type of task. This approach breaks down the original problem into individual, single-label learning tasks. First, a suitable binary classifier, such as logistic regression, is chosen to make simultaneous predictions of the binary targets. Each label is treated as an independent binary-classification task, and multiple instances of the base classifier are created, with each instance dedicated to a specific label. During training, the base classifier is trained on the input data, treating each label as a distinct target variable. This allows the classifier to learn how to predict the presence or absence of each label based on the input features. By adapting single-label classifiers to handle multi-label scenarios, we can make simultaneous predictions for multiple binary targets.

Similar to multi-label classification, multi-output regression aims to simultaneously predict multiple, real-valued output/target variables [23]. The formulation of the problem is slightly modified since target variables are considered to be continuous. The task is to create a learning model that will be able to simultaneously predict the target variables of new, incoming, unlabelled instances. The existing methods can be categorised as problem-transformation and algorithm-adaptation methods.

The main drawback of transforming the multi-output regression problem into single-output problems is that the targets are predicted independently, without maintaining the relationship between them. Therefore, to model the target dependencies, the regressor-chaining (RC) method is proposed. When training the RC a separate regression model is created for each random chain of targets. Let us assume the target variables are $Y=(y_{1},y_{2},...,y_{q})$. The first model of the RC predicts only the $y_{1}$. Then the subsequent models for $y_{j,j>1}$ are trained on the transformed dataset $D^{⁎}$ with the instance $E_{i}^{⁎}=(X_{i}^{⁎},Y_{i}^{⁎})$, where $X_{i}^{⁎}=(x_{i,1},x_{i,2},...x_{i,m},y_{i,1},...,y_{i,j-1})$ is the transformed input vector that contains the original input plus the actual values of all the previous targets in the chain. The current RC method is sensitive to the order of the chains. An improved version is the ensemble of regressor chains, where different RC models are trained on differently ordered chains. The same concept of chains can be applied to a classification problem as well.

Algorithm adaptation methods are able to capture all the dependencies and internal relationships between the target variables. Therefore, it is easier to interpret a single multi-output model than many single-output models. Some inherently multi-output models used in this research are linear regression, k-nearest neighbours, decision tree and random-forest regressor.

### 2.6 Machine-learning methods

The proposed approach is evaluated by comparing its predictive performance with several well-known ML algorithms:

• **Logistic regression (LR)**[24] is a supervised-learning algorithm commonly used for binary and multi-class classification tasks. It models the probability of a binary response variable based on one or more predictor variables, using a logistic or sigmoid function to map the input values to a probability output.
• **Linear regression (LNR)**[25] is a statistical modelling technique used to analyse the relationship between a dependent variable and one or more independent variables. LNR assumes a linear relationship between the dependent variable and the independent variables, seeking to find the best-fitting straight line or hyperplane through the data points. It aims to minimise the sum of the squared errors between the predicted and actual values of the dependent variable.
• **K-nearest neighbours (KNNs)**[26] is a versatile algorithm used for both classification and regression tasks. KNNs operates on the principle of finding the KNNs to a given data point in a feature space and classifying the data point based on the class labels of its neighbours. In the case of regression, the predicted value is often the average or median of the target values of the KNNs.
• **Decision tree (DT)**[27] is a tree-structured model that represents a sequence of decisions and their potential outcomes. It starts with a root node representing the initial decision and branches out into multiple nodes, each corresponding to a possible decision and its outcome. DT models are constructed by training on a labelled dataset, where the features and the corresponding labels are used to construct the tree. During the prediction, the decision tree traverses the nodes based on the features of the input instance, ultimately reaching a leaf node that represents the final prediction.
• **Random forest (RF)**[28] is an ensemble-learning method that builds multiple decision trees during the training and outputs the class that is the mode of the classes (classification) or the mean prediction (regression) of the individual trees. RF creates diverse decision trees by using bootstrap samples of the training data and random subsets of features at each split. The final prediction is made by aggregating the predictions of all the trees, resulting in a robust and accurate ensemble model.
• **Linear support-vector regression (LSVR)**[29] is a supervised-learning algorithm used for regression tasks. It is a variant of the Support Vector Machine (SVM) algorithm adapted for regression problems. The goal of LSVR is to find the best hyperplane that separates the input data points, while fitting as many data points as possible within a threshold margin. This threshold margin is defined by a hyperparameter called epsilon, which determines the margin of error allowed for the model.
• **Gradient-boosting regressor (GBR)**[30] is an ensemble method that combines several weak learners, often decision trees, into a strong learner. GB works by iteratively fitting decision trees to the residuals of the previous model, gradually reducing the overall prediction error. The final prediction is obtained by aggregating the predictions of all the weak learners, producing a powerful ensemble model.
• **Elastic net (EN)**[31] is a regularised linear-regression technique that combines both L1 (Lasso) and L2 (Ridge) regularisation. It is particularly useful for high-dimensional datasets where feature selection and regularisation are essential. EN simultaneously performs feature selection by driving irrelevant coefficients to zero (L1 regularisation) and prevents over-fitting by penalising the magnitude of the coefficients (L2 regularisation).
• **Stochastic Gradient Descent (SGD)**[32] is an iterative optimisation algorithm used to find the minimum of a cost function. It updates the model parameters using a random subset (mini-batch) of the training data, which makes it more scalable and computationally efficient, especially for large datasets.
• **Support Vector Regression (SVR)**[33] is a supervised machine-learning algorithm used for regression tasks. It shares the same principles as SVM for classification, aiming to find the best hyperplane that separates the input data points. However, in SVR, the goal is to fit as many data points as possible within a threshold margin, defined by a hyperparameter called epsilon. SVR can be employed with different kernel functions, such as linear, polynomial, or radial basis, to map the input data into higher-dimensional feature spaces where a linear-regression model can be used.
• **Bayesian ridge (BR)**[34] regression is a linear-regression model that employs Bayesian methods to estimate the regression coefficients. It provides a probabilistic model for both the response variable and the coefficients, allowing for the incorporation of prior knowledge or assumptions about the problem. The model optimises the hyperparameters of the prior distributions using a maximum-likelihood estimation and iteratively estimates the posterior distribution of the coefficients using a Bayesian updating scheme.

### 2.7 Model-quality assessment

Multi-label algorithms require distinct evaluation approaches; this is in contrast to single-label algorithms. In single-label tasks, instances are classified as either correct or incorrect, while in multi-label tasks they can be partially correct. For the evaluation, the average difference between the true and predicted values can be calculated using two methods: micro-averaging and macro-averaging. The metrics used in this study are described below. Micro-averaging aggregates the results by summing the true positives, false positives and false negatives. Macro-averaging calculates the average of a specific metric. Equations (4) and (5) show the micro-average, while equations (6) and (7) show the macro-average of precision and recall. In the equations *H* represents the trained classifier, *D* is the testing dataset and $y_{i}\in Y,i=1,...q$ represents the multi-label target variables.

(4) $$Prc^{micro}(H,D)=\frac{\sum_{y_{y}\in Y}TPs(y_{i})}{\sum_{y_{i}\in Y}TPs(y_{i})+FPs(y_{i})}$$

(5) $$Rcl^{micro}(H,D)=\frac{\sum_{y_{i}\in Y}TPs(y_{i})}{\sum_{y_{i}\in Y}TPs(y_{i})+FNs(y_{i})}$$

(6) $$Prc^{macro}(H,D)=\frac{\sum_{y_{i}\in Y}Prc(D,y_{i})}{\left|Y\right|}$$

(7) $$Rcl^{macro}(H,D)=\frac{\sum_{y_{i}\in Y}Rcl(D,y_{i})}{\left|Y\right|}$$

To determine the effectiveness of a regression model, several commonly used evaluation metrics are utilised. These metrics include the negative mean-squared error (MSE), the mean-absolute error (MAE), the coefficient of determination (R2) and the root-mean-squared error (RMSE). When applying these metrics to a multi-output regression problem, it is necessary to calculate the errors for each target variable separately and then sum or average them. This ensures that each target variable's performance is evaluated independently and accurately.

It should be noted that multi-output ML does not provide uniform, deterministic outputs like classical ML. Consequently, a single input can lead to multiple outputs. To distinguish or validate these outputs from the perspective of a decision maker, it might be necessary to rerun simulations for fine tuning. Fortunately, this process is infrequently utilised and is not overly complex or time consuming. However, it is worth noting that this issue does not impact on the computation of MAE and other metrics.

MAE (Equation (8)) measures the average absolute difference between the predicted and true values. It quantifies the average magnitude of the errors:

(8) $$MAE=\frac{\sum_{i=1}^{n}|Y_{pred}-Y_{true}|}{N}$$

MSE measures the average squared difference between the predicted and true values (Equation (9)). Also, it emphasises larger errors due to the squared term. The negative sign indicates that the lower values of MSE correspond to better performance of the model. By negating the MSE, we align it with the convention where smaller values indicate better accuracy.

(9) $$negMSE=-\frac{\sum_{i=1}^{n}{\left(Y_{pred}-Y_{true}\right)}^{2}}{N}$$

R2 is a statistical measure that represents the proportion of the variance in the dependent variable that can be explained by the independent variables (Equation (10)). It indicates the goodness of fit of the regression model.

(10) $$R2=1-\frac{\sum_{i=1}^{n}{\left(Y_{true}-Y_{pred}\right)}^{2}}{\sum_{i=1}^{n}{\left(Y_{true}-{\overset{¯}{Y}}_{true}\right)}^{2}}$$

RMSE is a variant of MSE that measures the square root of the average squared difference between the predicted and true values (Equation (11)). It is used to quantify the typical magnitude of the errors in the predictions:

(11) $$RMSE=\sqrt{\frac{\sum_{i=1}^{n}{\left(Y_{pred}-Y_{true}\right)}^{2}}{N}}$$

In the above equations (8), (9), (10), (11), $Y_{\text{pred}}^{\left(i\right)}$ represents the predicted value for the *i*th instance, $Y_{\text{true}}^{\left(i\right)}$ represents the true value for the *i*th instance, *N* represents the total number of instances, and ${\overset{¯}{Y}}_{\text{true}}$ represents the mean of the true values.

The overall performance of the models is calculated as the mean value of the evaluation metrics for each target variable $y_{i}\in Y$:

(12) $$M_{mean}=\frac{\sum_{i=1}^{q}My_{i}}{q}$$

In equation (12), *q* represents the number of target variables and *M* refers to a specific metric.

### 2.8 Statistical analysis

In addition to evaluating regression models using metrics like negative MSE, MAE, R2, and RMSE, a p-test analysis is conducted to compare the best-performing method with the others. This analysis provides insights into the statistical significance of the results, i.e., whether the observed differences between the models are likely due to chance or genuine variations in performance.

The significance level (*α*) serves as a predetermined threshold to determine the statistical significance. Common levels include 0.05 (5%) and 0.01 (1%). If the p-value is lower than the significance level (p<α), it indicates statistically significant results.

## 3 Results

### 3.1 Data and scenarios

In this study the city of Bilbao provided the data used as the input for the approach. This includes information about the city map and the schedules for the public transport, as well as demographic data from the EU Statistics on Income and Living Conditions (EU-SILC) survey covering 10% of the population [35]. To generate the travel demand, origin-destination matrices were used to assign the activity locations.

A requirement for ML is having enough samples to train the models. Hence, various scenarios were simulated with the previously described methods. The scenarios demonstrate the implementation of mobility policies in a city. Although a single scenario could entail multiple policies and vice versa, we limited our experiments to each scenario being exactly one policy, along with a default scenario that implements no policy, i.e., it represents the current mobility situation in the city.

**Figure 4:**
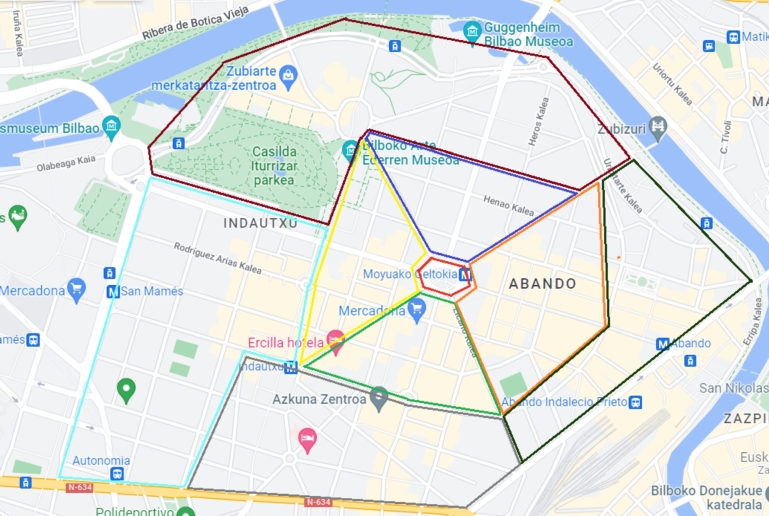
Nine city areas for closure.

These scenarios are specific to the city, for example, closing a certain district in a city cannot be easily transferred to another city that does not have that district. Thus, we defined a set of scenarios that are specific to Bilbao, our target city in the study. The selected mobility policies involve closing the Moyua square in the city centre for traffic at a specific time of day and closing the surrounding areas in different combinations. Nine non-overlapping areas were considered, as shown in Fig. 4. We simulated the closure of these nine areas individually and then combined them in different ways. When combining the areas, i.e., closing multiple areas at once, the central area (Moyua square) was included in most instances, and the closed areas were connected to each other. This resulted in 33 combinations of area closures.

**Figure 5:**
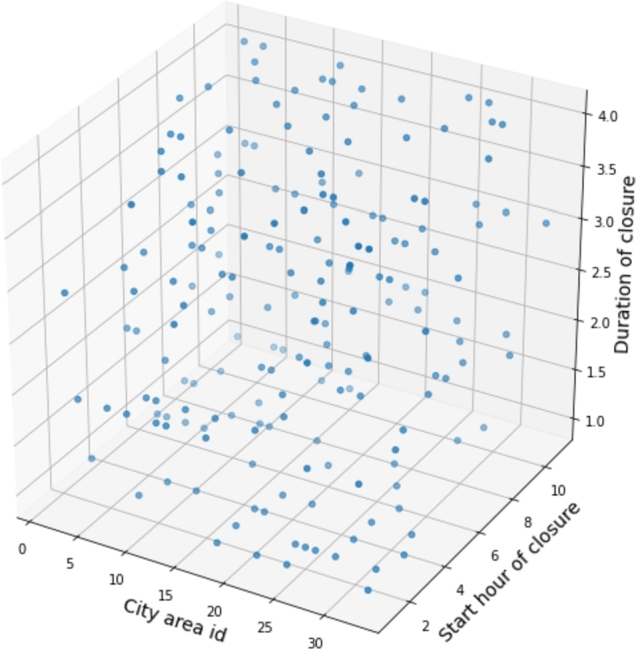
Simulated scenarios, selected with the LHS sampling.

In addition to closing areas in the city, the scenarios also specify the start time and the duration of the closure. The start times range from 7 am to 5 pm, with closures lasting 1 to 4 hours (in 1-hour steps), resulting in 1452 possible scenarios. While simulating all the combinations would deliver the best mobility policy, it is impractical because of each simulation's 3-hour computing demand. Thus, a subset was chosen for the simulation using the Latin hypercube sampling (LHS) method [36] for parameter values. Fig. 5 shows LHS-generated samples, encompassing closed city areas, starting hour of the closure, and the duration.

### 3.2 Implementing machine learning for the mobility-policy recommendations

Our approach employs ML to construct a decision model that predicts the most appropriate policy actions aligned with the objectives of the decision makers. Developing this model necessitates a dataset of policy actions and the resulting outcomes, like lower levels of pollution and shorter travelling times. This data is acquired through a microscopic traffic simulator. The traffic simulation requires several models, such as population and travel-demand models, and a calibration process to accurately imitate the behaviour of the traffic in the city. These steps are based on actual traffic data and are outlined in the following text.

**Figure 6:**
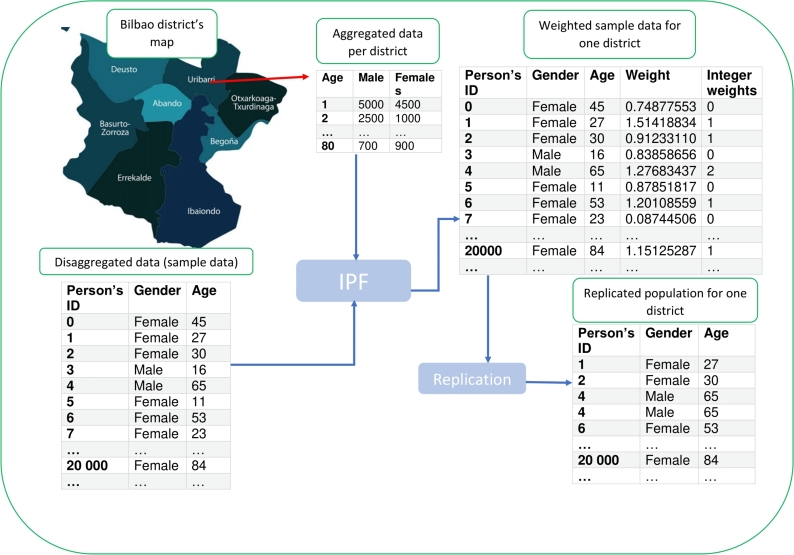
Synthetic-population generation steps.

The process of generating a synthetic population is illustrated in Fig. 6. The IPF algorithm takes the aggregated and dis-aggregated datasets as the input and produces weights, representing the required replication instances of distinct individuals, while maintaining the marginal distributions of a district. The process is repeated for each district. See [19] for more info about the synthetic-reconstruction approach. The resulting synthetic population has around 300,000 individuals, complete with home location, age and gender attributes. The travel-demand model incorporates activity locations with population data from the synthetic-population model to generate the necessary travel demand. It uses an origin-destination (OD) matrix from the target city. OD matrices depict people's movements, with each cell representing a trip from an origin to a destination. An example of the travel data produced by the travel-demand model is presented in Table 1.

**Table 1:** An example of the synthetic data generated from the travel-demand model.

Persons' groups	Age	Activities	Transport mode
young students	<16	home, education	public transport, bicycle, walk

young students	≥16,<18	home, education	car, public transport, bicycle, walk

university students	≥18,<24	home, education, work, leisure, shopping, other	car, public transport, bicycle, walk

regular workers	≥24,<65	home, work, leisure, shopping, other	car, public transport, bicycle, walk

delivery service workers	≥24,<65	home, work	bicycle

elderly	≥65	home, leisure, shopping, other	car, public transport, bicycle, walk

MATSim conducted simulations of the given input files for each scenario. A total of 192 simulations were performed, each encompassing 200 optimisation iterations, a configuration established in previous research [37] for achieving equilibrium. The average duration for one simulation was roughly 3 hours on a PC. The total simulation time was 576 hours, equivalent to more than 24 days on a PC or more than a week on three dedicated servers.

**Figure 7:**
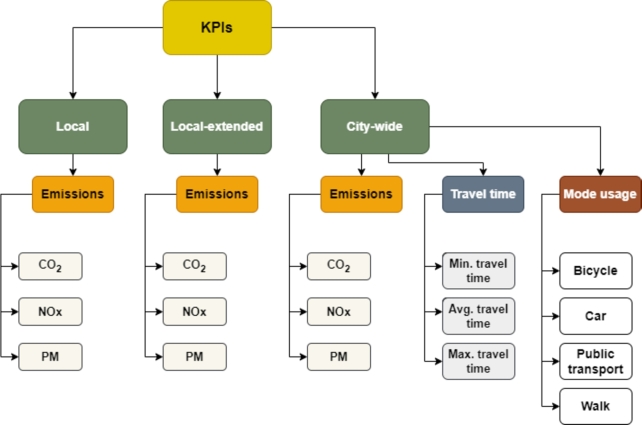
KPIs.

In collaboration with the city decision makers, a set of KPIs was defined to measure the emissions and traffic, shown in Fig. 7. The KPIs are calculated at three levels: local, local-extended, and city-wide. Local KPIs are calculated for policy-affected areas, for example, closed areas like Moyua square. The local-extended level includes surrounding areas influenced by the policy, and city-wide KPIs cover the entire city. Emissions are calculated for each level, and traffic KPIs, including travel times and mode usage, are calculated for the entire city. Most of the KPIs are calculated from the MATSim's output, while the emissions are modelled with an additional MATSim module [38] that uses the HBEFA (Handbook on Emission Factors for Road Transport) database [39].

Once the selected scenarios are simulated and the desired KPIs are calculated, a set of features and target variables needs to be defined to train the ML models.

All the features represent percentage alterations relative to the baseline scenario, which mirrors the current urban-mobility status devoid of policy adjustments. Target variables, denoting mobility policies, encompass start hour, duration and closed areas. These features and target variables are outlined in Table 2, providing insight into the input data features. The specific rules for a given simulation run are encapsulated in a single row within the training input matrix. The semantics is as follows: if we want to achieve a specific improvement in a city, e.g., a specific decrease of $CO_{2}$, NOx and similar, then we need to close a specific area with a specific start time and duration of closure.

**Table 2:** Features and target variables.

Features				Target variables
local	local-extended	city-wide	simulation	
CO_2_	CO_2_	CO_2_	*bicycle usage*	*start time of closure*
NOx	NOx	NOx	*car usage*e	*duration of closure*
PM	PM	PM	*walk usage*	*closed areas*
			*pt usage*	
			*min travel time*	
			*avg travel time*	
			*max travel time*	

The task of predicting the closure schedule and the affected areas is divided into two parts due to the varied target variables. In the first part, multi-output regression models are used to predict the start hour and the duration of the closures, while a binary representation of nine areas is used to indicate which areas are closed. Ten regression algorithms were tested: LNR, KNN, DT, RF, LSVR, GBR, EN, SGD, SVR and BR. The first four algorithms support multi-output regression natively, while the rest are trained through problem-transformation techniques.

According to the MAE evaluation, GBR demonstrated the best performance with an average error of 1.50 in the k-cross-fold validation. Additionally, MSE, RMSE, and R2 metrics were computed for further assessment.

To assess the significance of the superiority of GBR over the other models, a t-test analysis was carried out. The evaluation metrics for all 10 models, along with corresponding p-values relative to GBR, are shown in Table 3. Consequently, the p-value indicates the presence of statistically significant distinctions between each model and GBR.

**Table 3:** Evaluation Metrics and P-values (*α* = 0.05).

Algorithm	MAE		MSE		RMSE		R2	
	mean	p-value	mean	p-value	mean	p-value	mean	p-value
GBR	1.50	/	-5.43	/	1.36	/	0.31	/
LR	1.51	0.69	-5.06	0.09	1.35	0.32	0.33	0.41
RF	1.52	0.46	-5.32	0.30	1.35	0.03	0.33	0.10
SGD	1.55	0.50	-5.03	0.09	1.37	0.38	0.31	0.63
LSVR	1.55	0.14	-5.30	0.50	1.37	0.56	0.29	0.44
BR	1.58	0.01	-5.21	0.20	1.36	0.73	0.32	0.56
SVR	1.59	0.00	-5.32	0.56	1.38	0.05	0.24	0.01
EN	1.75	0.00	-5.49	0.74	1.42	0.00	0.09	0.00
KNN	1.75	0.00	-6.05	0.01	1.45	0.00	0.03	0.00
DT	1.99	0.00	-10.37	0.00	1.61	0.00	-0.35	0.00

Based on the results, LR, RF, SGD, and LSVR perform similarly to GBR since their p-values exceed the significance level. On the other hand, BR, SVR, EN, KNN, and DT show statistically significant differences when compared to GBR.

To further estimate the GBR model's quality, an additional t-test analysis was conducted, comparing the simulation results (ground truth) with the predictions derived from the GBR model, i.e., the start hour and duration. The resulting p-values were 0.9826 and 0.9602, respectively. Hence, based on the t-test findings, we can confidently ascertain that the GBR model adequately predicts both the start hour and the duration, evidenced by its close concurrence with the ground-truth simulations.

**Figure 8:**
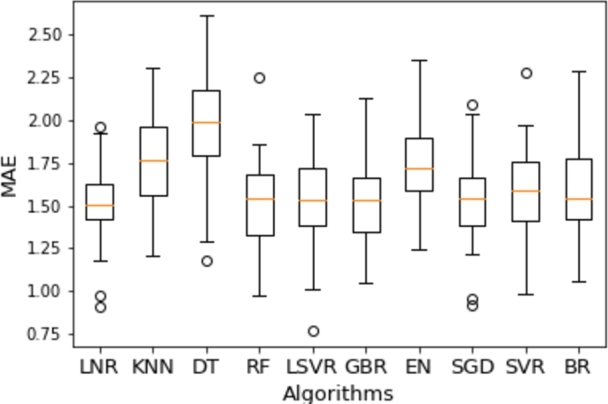
Comparison of methods.

The results of all the regression models are shown in Fig. 8. The boxplot shows how the scores of each fold are spread out. The orange line in each box shows the mean value of the MAE over all folds. Linear regression proved to be the best, with a mean MAE value of 1.514 and a standard deviation of 0.230. In other words, the model can predict the start hour and the duration of closure, with an error of 1.5 hours based on a given input of KPI values (Table 2).

**Figure 9:**
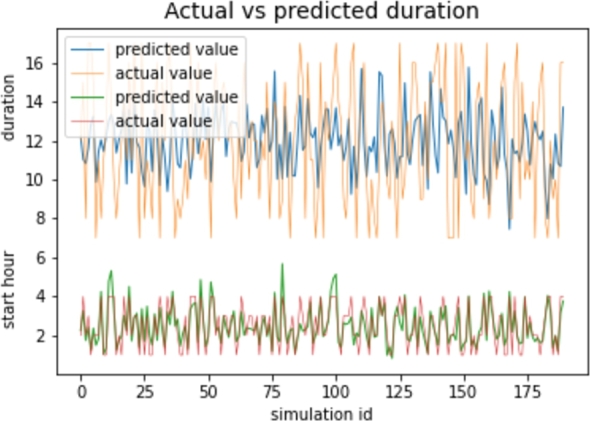
Actual vs predicted start hour and duration.

Fig. 9 shows the difference between the predicted and real values using linear regression and k-fold cross-validation for duration and start hour.

**Figure 10:**
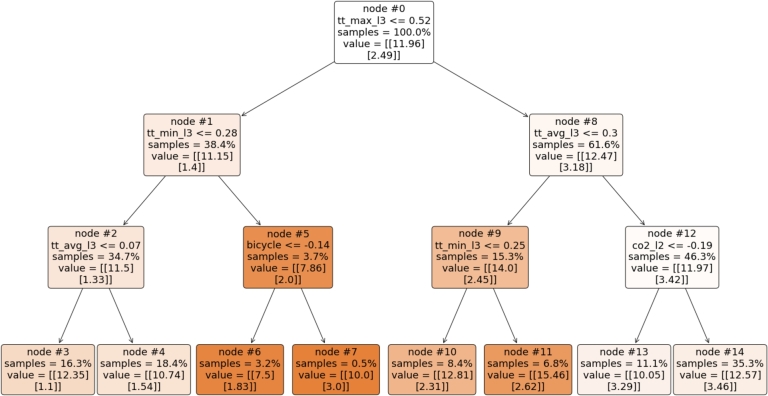
Regression tree.

A regression tree, shown in Fig. 10, enhances the transparency by revealing the relationships between the input features and the transport outcomes. Initially, the tree uses $tt_{\text{max}\_\text{l3}}$ (maximum travel time on level-3 roads - the entire city) as the primary splitting criterion, i.e., as one of the most important features. It then branches based on $tt_{\text{min}\_\text{l3}}$ and the presence of bicycles. The tree effectively showcases how these factors influence the travel time and the emissions outcomes. Additionally, $tt_{\text{avg}\_\text{l3}}$ plays a pivotal role in further splitting, resulting in distinct predictions for various scenarios.

In the second part, multi-output, binary-classification models are used to predict which areas will be closed. The predicted start hour and duration of the closures are added to the feature vectors to create models for predicting the closed areas. This task is also supported by problem-transformation techniques, and logistic regression is used to predict each area individually.

**Figure 11:**
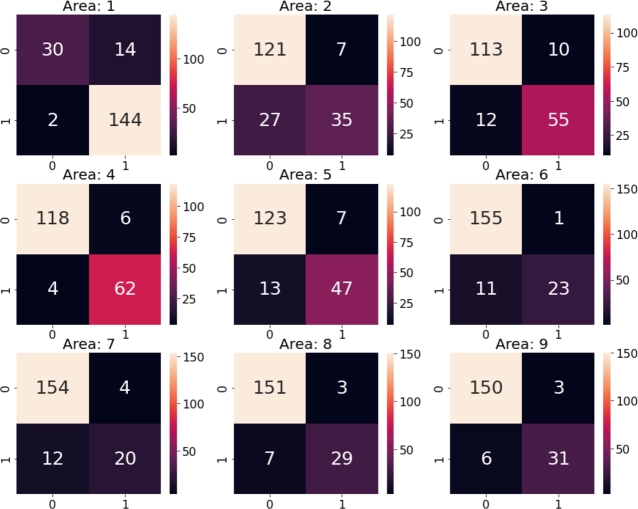
Confusion matrices for each area of closure.

Fig. 11 shows nine confusion matrices, each for one area. The closure of the areas is treated as a negative event, while the positive event represents instances where no modification in the current area is applied. For example, area 2 was opened 128 times, correctly classified 121 times and incorrectly 7 times.

**Figure 12:**
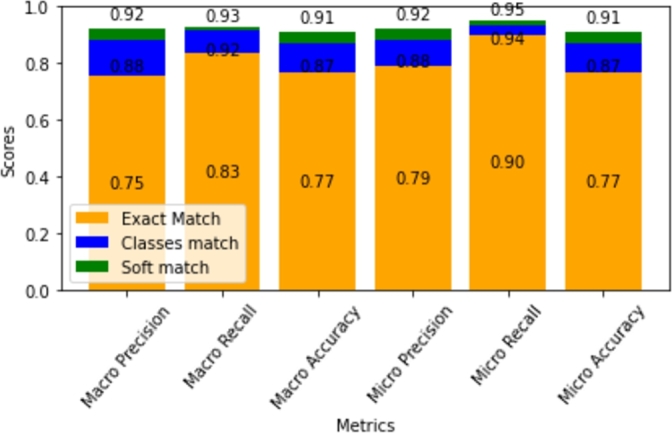
Comparison of evaluation metrics.

The overall performance of the ML model is shown in Fig. 12. The model achieved 90% recall score/true positive rate and 77% accuracy with exact matches of the features and labels (orange). To comprehensively assess the model's effectiveness, two additional approaches were explored.

The challenge with exact matching is that an erroneous prediction for even a single area causes the misclassification of the entire instance, disregarding the potential for enhanced outcomes through a combination of predicted closures, leading to reduced $CO_{2}$ emissions and shorter travel times. To address this, an alternative approach incorporated the Euclidean distance between the predicted and actual values. Introducing a defined threshold enabled a distinction between the accurate and inaccurate classifications for each instance. The bar plot, highlighting results in blue, exhibits a notable accuracy enhancement, elevating it from 77% to 87%. The meaning of this modification is that a solution close enough is probably good enough, although not an exact match. Potentially, fine tuning with actual simulations is needed in this case.

Furthermore, a third approach was employed, which entailed exploring feature vectors associated with similar policy outcomes, closures, and durations through Euclidean distance measurements. If the predicted class vector closely aligned with any of these chosen feature-vector classes, the prediction was deemed accurate. This approach is indicated by green on the bar plot, achieving 91% without tuning parameters. The implication is that similar or even better outcomes are achievable through similar policies, which align with the objectives set by a decision maker. Note that there were no hand- or Auto-Skealrn-tuning of the parameters.

Employing these three distinct approaches provides better insights into the model's predictive abilities, enabling better-informed decisions regarding the best areas for closure.

## 4 Discussions and conclusions

The primary aim of the URBANITE project was to create an open-source, advanced ecosystem that allows for the testing of mobility policies in any smart city. The system simulates human behaviour so as to better encapsulate the impacts of different mobility strategies and policies, with the ultimate objective of facilitating the creation of more sustainable cities. While harder to design and implement than predicted, the system is operational and freely accessible. The URBANITE open-data, open-source framework consists of around 2 million lines of code, running in separate environments and different operating systems. Each stand-alone model is available as open-source and the whole system can be tested as presented in the supplementary materials.

As part of the system, several novel models are introduced, most of them founded on ML and AI. The multi-output ML module presented in this paper starts by collecting city-specific data that enable the generation of a synthetic population and a travel-demand model. These two models provide the input for simulations using the MATSim traffic simulator. By conducting multiple simulations, an extensive data set can be generated that can then be used to discern patterns and the relationships between different variables with ML. In addition to the statistical data, the city-specific KPIs are calculated for the city, offering valuable insights into various aspects of the city's mobility system.

The main research hypothesis, i.e., that multi-output ML methods can be applied to the simulations' output to automate the decision-making process and expedite the optimisation process, proved accurate. The multi-output module in the URBANITE project is unique as it can help explicate the intricate relationships between factors such as traffic patterns, travel behaviour, and energy consumption in a novel way. This helps city planners and decision makers to make informed decisions about the city's future. The ML approach provides numerous advantages, including transparency. For instance, a regression tree shown in Fig. 10 helps us to understand what most influences the decrease in pollution in a structured manner.

To the best of our knowledge, this study is the first to address policy testing in a real city using multi-label ML. While the approach is general, it has so far only been applied to Bilbao as a demonstrative prototype. Plans to include other major European cities collaborating in the URBANITE project are underway and could be implemented in a few months.

Apart from the new knowledge and patterns discovered, the ML module provides a considerable speed-up, reducing the time from 3 hours for one simulation to just 10 seconds for a new simulation with the learned ML module. However, while learning the ML required 23 days for 192 simulations, around 1452 simulations would need approximately 6 months in the case of Bilbao on a PC. This time improvement allows for the nearly interactive use of ML modules for key decision makers, replicating the performance of simulations with a high degree of similarity. Since the problem has an exponential time demand, the time needed for all the simulations would soon grow beyond any computer's capabilities, yet the time needed for one response from a ML model would remain about the same. It is important to note that this significant time improvement primarily benefits key city decision makers. For computer developers, it demands more effort than simply running a simulator.

It is also important to note that the quality of the models is contingent on the quality of the data used to train them. Therefore, it is critical to employ statistical methods to evaluate the models' accuracy and reliability. This can be achieved by comparing the predictions of the models to actual data, or by using other validation techniques.

Another limitation of the ML approach presented here is that it requires staff who are proficient in computing and ML. For instance, another ML module in URBANITE connects the simulation outputs to Orange [40] and facilitates programming through a graphical input. Although it offers dozens of freely available methods, it does not include multi-output ML. This study is focused on an advanced ML approach, requiring more skills and effort, but offering a more advanced viewpoint to decision makers.

As regards future work, we aim to extend the application of this ML approach to other pilot cities within the URBANITE project. There is also a need to improve the system to become more user-oriented and user-friendly; however, programming-trained staff will still be needed to adapt the module for a specific city. Additionally, we plan to test more complex models to enhance their quality and improve the predictions. For instance, we could explore the use of deep-learning techniques such as convolutional neural networks or recurrent neural networks to capture the complex relationships between features and target variables. Moreover, additional data sources could be incorporated, such as weather conditions, event schedules and road-network information, to further enhance the performance of the models. By exploring more advanced models, we aim to continually improve the models' accuracy and efficiency, leading to better decision making.

In practical terms it would be beneficial to upgrade the overall URBANITE research prototype into a user-friendly, open-source system for all European cities to use. In the current project, one of the core problems was the scalability, i.e., the size of the software and the compatibility between various sub-modules. Once these issues were resolved, the future focus should turn to improved user-friendliness, functionality, new modules, and of course adding a GPT-like interface for communication with the whole system. The ML module already proved its power, but there is lots of room for improvement, e.g., to integrate several ML sub-modules into one AI system capable of proposing improvements to the city's mobility on its own, i.e., proactively, given general directions and policies.

## CRediT authorship contribution statement

Miljana Shulajkovska: Conceived and designed the experiments; Performed the experiments; Analysed and interpreted the data; Contributed reagents, materials, analysis tools or data; Wrote the paper. Maj Smerkol: Analysed and interpreted the data; Contributed reagents, materials, analysis tools or data. Erik Dovgan; Matjaž Gams: Conceived and designed the experiments; Analysed and interpreted the data; Contributed reagents, materials, analysis tools or data; Wrote the paper.

## Additional information

No additional information is available for this paper.

## Declaration of Competing Interest

The authors declare the following financial interests/personal relationships which may be considered as potential competing interests:

Miljana Shulajkovska reports financial support was provided by Horizon 2020 European Innovation Council Fast Track to Innovation.

## Data Availability

Data associated with this study has been deposited at https://repo.ijs.si/urbanite/ml-mobility-proposal/-/tree/master.

## References

[1] UN DESA (2019).

[2] Dameri R.P. (2013). Searching for smart city definition: a comprehensive proposal. Int. J. Comput. Technol..

[3] Washburn D., Sindhu U., Balaouras S., Dines R.A., Hayes N., Nelson L.E. (2009). Helping cios understand “smart city” initiatives. Growth.

[4] Anthopoulos L.G. (2015). Transforming City Governments for Successful Smart Cities.

[5] Bibri S.E., Krogstie J. (2017). Smart sustainable cities of the future: an extensive interdisciplinary literature review. Sustain. Cities Soc..

[6] Bifulco F., Tregua M., Amitrano C.C., D'Auria A. (2016). Ict and sustainability in smart cities management. Int. J. Public Sect. Manag..

[7] Benevolo C., Dameri R.P., D'auria B. (2016). Empowering Organizations.

[8] Dameri R.P. (2017). Smart City Implementation.

[9] Melkonyan A., Gruchmann T., Lohmar F., Bleischwitz R. (2022). Decision support for sustainable urban mobility: a case study of the Rhine-Ruhr area. Sustain. Cities Soc..

[10] Shahidehpour M., Li Z., Ganji M. (2018). Smart cities for a sustainable urbanization: illuminating the need for establishing smart urban infrastructures. IEEE Electrif. Mag..

[11] Kaczmarek I., Iwaniak A., Świetlicka A., Piwowarczyk M., Nadolny A. (2022). A machine learning approach for integration of spatial development plans based on natural language processing. Sustain. Cities Soc..

[12] TECNALIA (2023). Urbanite. https://urbanite-project.eu.

[13] Shulajkovska M., Noveski G., Smerkol M., Grabnar J., Dovgan E., Gams M. (2023). EU smart cities: towards a new framework of urban digital transformation. Informatica.

[14] Smerkol M., Sulajkovska M., Dovgan E., Gams M. (2021).

[15] Dovgan E., Smerkol M., Sulajkovska M., Gams M. (2021).

[16] Smerkol M., Shulajkovska M., Dovgan E., Gams M. (2021).

[17] Sulajkovska M., Smerkol M., Dovgan E., Gams M. (2021).

[18] Huynh N., Barthelemy J., Perez P. (2016).

[19] Lovelace R., Dumont M., Ellison R., Založnik M. (2017).

[20] A. Horni, K. Nagel, K.W. Axhausen, Introducing MATSim, Ubiquity Press, pp. 3–8.

[21] K. Nagel, B. Kickhöfer, A. Horni, D. Charypar, A Closer Look at Scoring, Ubiquity Press, pp. 23–34.

[22] Cherman E., Monard M.-C., Metz J. (2011). Multi-label problem transformation methods: a case study. CLEI Electron. J..

[23] Borchani H., Varando G., Bielza C., Larrañaga P. (2015). A survey on multi-output regression. WIREs Data Min. Knowl. Discov..

[24] Das A. (2021). Encyclopedia of Quality of Life and Well-Being Research.

[25] Maulud D., Abdulazeez A.M. (2020). A review on linear regression comprehensive in machine learning. J. Appl. Sci. Technol. Trends.

[26] Abu Alfeilat H.A., Hassanat A.B., Lasassmeh O., Tarawneh A.S., Alhasanat M.B., Eyal Salman H.S., Prasath V.S. (2019). Effects of distance measure choice on k-nearest neighbor classifier performance: a review. Big Data.

[27] Charbuty B., Abdulazeez A. (2021). Classification based on decision tree algorithm for machine learning. Journal of Applied Science and Technology Trends.

[28] Schonlau M., Zou R.Y. (2020). The random forest algorithm for statistical learning. Stata J..

[29] Huang H., Wei X., Zhou Y. (2022). An overview on twin support vector regression. Neurocomputing.

[30] Bentéjac C., Csörgő A., Martínez-Muñoz G. (2021). A comparative analysis of gradient boosting algorithms. Artif. Intell. Rev..

[31] Amini F., Hu G. (2021). A two-layer feature selection method using genetic algorithm and elastic net. Expert Syst. Appl..

[32] Netrapalli P. (2019). Stochastic gradient descent and its variants in machine learning. J. Indian Inst. Sci..

[33] Pisner D.A., Schnyer D.M. (2020). Machine Learning.

[34] Michimae H., Emura T. (2022). Bayesian ridge estimators based on copula-based joint prior distributions for regression coefficients. Comput. Stat..

[35] Eurostat (2020).

[36] Loh W.-L. (1996). On Latin hypercube sampling. Ann. Stat..

[37] Llorca C., Moeckel R. (2019). Effects of scaling down the population for agent-based traffic simulations. Proc. Comput. Sci..

[38] Kickhöfer B. (2016).

[39] Keller M., Hausberger S., Matzer C., Wüthrich P., Notter B. (2017). Hbefa version 3.3, background documentation. Berne.

